# The infiltration risk prediction models by logistic regression for ground-glass pulmonary nodules: a systematic review and meta-analysis

**DOI:** 10.3389/fonc.2024.1477730

**Published:** 2025-01-29

**Authors:** Mengqian Li, Xiaomei Zhang, Yuxin Lai, Yunlong Sun, Tianshu Yang, Xinlei Tan

**Affiliations:** ^1^ Department of Internal Medicine of Chinese Medicine, Beijing University of Chinese Medicine, Beijing, China; ^2^ Department of Pulmonary Nodules and Chest Diseases Center, Dongfang Hospital, Beijing University of Chinese Medicine, Beijing, China

**Keywords:** infiltration, independent risk factors, logistic regression, prediction model, systematic review and meta-analysis, ground glass pulmonary nodules

## Abstract

**Methods:**

CNKI, Wanfang, VIP, Sinomed, Pubmed, Web of Science, Embase, and other databases were searched. The retrieval time was from the establishment of the database to January 31, 2024. We included all predictive models for the invasion of ground-glass pulmonary nodules established. The modeling group was patients with a pathological diagnosis of ground-glass pulmonary nodules. Two researchers screened the literature, established an Excel table for information extraction, used SPSS 25.0 to perform frequency statistics of each independent risk factor, and used Revman 5.4 software for meta-analysis.

**Results:**

A total of 29 articles were included, involving 30 independent risk factors, with a cumulative frequency of 99 times. There were 16 risk factors with a frequency of ≥2 times, a total of 85 times, accounting for 85.86%. The meta-analysis showed the following: average CT value (MD = 75.57 HU, 95%CI: 44.40–106.75), maximum diameter (MD = 4.99 mm, 95%CI: 4.22–5.77), vascular convergence sign (OR = 11.16, 95%CI: 6.71–18.56), lobulation sign (OR = 3.80, 95%CI: 1.59–9.09), average diameter (MD = 4.46 mm, 95%CI: 3.44–5.48), maximum CT value (MD = 112.52 HU, 95%CI: 8.08–216.96), spiculation sign (OR = 4.46, 95%CI: 2.03–9.81), volume (MD = 1,069.37 mm^3^, 95%CI: 1,025.75–1,112.99), vacuole sign (OR = 6.15, 95%CI: 2.70–14.01), CTR ≥0.5 (OR = 7.24, 95%CI: 3.35–15.65), vascular type [types III and IV] (OR = 13.62, 95%CI: 8.85–20.94), pleural indentation (OR = 6.92, 95%CI: 2.69–17.82), age (MD = 4.18years, 95%CI: 1.70–6.65), and mGGN (OR = 3.62, 95%CI: 2.36–5.56) were risk factors for infiltration of ground-glass nodules. The overall risk of bias in the methodological quality evaluation of the included studies was small, and the AUC value of the model was 0.736–0.977.

**Conclusion:**

The included model has a good predictive performance for the invasion of ground-glass nodules. The independent risk factors included in the model can help medical workers to identify the high-risk groups of invasive lung cancer in ground-glass nodules in time and improve the prognosis.

## 1 Introduction

Lung cancer is a malignant tumor with high morbidity and mortality. The 2020 GLOBOCAN data show that lung cancer ranks second in the world’s most common cancers (1). Early lung cancer is more common in the form of ground-glass pulmonary nodules (GGNs) (2). GGNs mostly experienced the evolution of AAH to AIS to MIA and to IAC, and the “three stages” of lung adenocarcinoma—AAH, MIA, and IAC—can also exist in multiple GGNs of the same patient (3). Ground-glass pulmonary nodules refer to abnormal density shadows with a diameter of ≤3 cm in the lung, the nodules do not completely cover the lung parenchyma, and the internal blood vessels, bronchi, and other structures are clearly displayed (4). Studies have shown that 95.5% of lung cancer shows ground-glass shadows (5). GGNs can be divided into pure ground-glass nodules (pure GGN, pGGN) and mixed ground-glass nodules (mixed GGN, mGGN) according to the imaging findings (6). The degree of malignancy and the possibility of invasive growth of mGGN are higher (7), but it is difficult to strictly distinguish the two.

The 2021 WHO classification of thoracic tumors (5th edition) (8) divided lung tumors into glandular precursor lesions [atypical adenomatous hyperplasia (AAH) and adenocarcinoma *in situ* (AIS)], microinvasive adenocarcinoma (MIA), and invasive adenocarcinoma (IAC). Among them, the 5-year disease-free survival rate of AAH, AIS, and MIA can reach 100%, while the 5-year disease-free survival rate of IAC is only 40%–85% (9, 10). In AAH, AIS, and MIA, segmentectomy without lymph node dissection is usually used, and they are classified as non-invasive indolent lung cancer, while IAC mostly requires total lobectomy and belongs to invasive lung cancer (11). Persistent ground-glass pulmonary nodules often experience the evolution of AAH to AIS to MIA and to IAC, and about 75% of persistent GGNs can be attributed to AIS or MIA (12). In recent years, risk prediction models of ground-glass pulmonary nodules infiltration have emerged in an endless stream. With the help of the GGN infiltration risk prediction model and related independent risk factors, early identification of IAC high-risk groups is of great significance to prevent IAC, improve the prognosis, and select appropriate surgical methods. The most common of these prediction models is logistic regression model. Therefore, this study intends to systematically evaluate and perform a meta-analysis of the GGN infiltration risk prediction logistic regression model to screen the influencing factors affecting GGN infiltration in order to provide a strong basis for the early clinical diagnosis of invasive GGNs and selection of appropriate treatment options.

## 2 Methods

### 2.1 Literature search

We used computers to perform research in CNKI, Wanfang, VIP, Sinomed, Pubmed, Web of Science, Embase, and other databases. The search time was from the establishment of the database to January 31, 2024. All of the prediction models of the risk of infiltration of ground-glass pulmonary nodules were comprehensively searched. The Chinese search terms used were as follows: pulmonary ground-glass nodules/ground-glass pulmonary nodules, infiltration/invasion, prediction model. The English database search terms included the following: ground-glass pulmonary nodules/pulmonary ground-glass nodules, invasion/infiltration, prediction model.

### 2.2 Inclusion criteria

(1) The research on the prediction model of the invasive risk of ground-glass pulmonary nodules included those that were published at home and abroad, and ground-glass pulmonary nodules were diagnosed according to CT; (2) The study type was retrospective study or prospective study; (3) The subjects were patients with pathologically diagnosed ground-glass pulmonary nodules, including non-invasive (AAH + AIS + MIA or AIS + MIA) and invasive (IAC) patients; (4) Nodule diameter is ≤3 cm; (5) The model is logistic regression model; (6) The literature is complete, including independent predictors, number of modeling cases, distribution of noninvasive and invasive groups, gender distribution, AUC value, sensitivity, specificity, etc.

### 2.3 Exclusion criteria

The following were excluded: (1) abstracts, reviews, letters, patents, conferences/dissertations, and nonclinical studies; (2) literatures with incomplete data, unmodeled or repeated modeling, comparison or verification with existing models, and poor research quality; (3) radiomics, random forest, histogram model, etc.; (4) those with independent risk factors <2 and unclear description of model information; and (5) language is non-Chinese and English literature.

### 2.4 Literature screening and data extraction

Two researchers screened the literature, extracted the data, and checked them together. If there were differences of opinion, they would discuss and decide with the third researcher. The content of data extraction included the following: title, first author, start and end time of study, study area, diameter of nodules included, sample size of modeling, number of non-invasive and invasive cases, gender, independent predictors and statistics (mean ± standard deviation or quartile of continuous variable extraction [save two decimals], frequency of categorical variable extraction), modeling method, AUC value, sensitivity, specificity, etc.

### 2.5 Name normalization and data transformation of independent predictor factors

The names of independent predictors included in each model are standardized in combination with the full text of the literature—for example, the maximum diameter, the long diameter, and other uniform specifications are the maximum diameter; the average diameter, the average value of the length diameter, and short diameter are the average diameter; vascular abnormality sign, internal vascular sign, vascular morphology in nodules, vascular convergence, and other unified norms were vascular convergence sign; bronchial inflation sign, air bronchial anomaly sign, bronchiole sign, and other unified norms were air bronchial sign; pleural traction and pleural retraction are unified specifications for pleural indentation sign; and the average CT attenuation and the average CT value of the ground-glass component are uniformly standardized as the average CT value. The data that the independent predictors are continuous variables and only provide quartiles in the original literature are transformed, and the mean and standard deviation are calculated. The transformation method references the research of Luo’s study (13) and Wan’s study (14).

### 2.6 Literature quality assessment

The methodology and quality of the final included literature were evaluated with the help of the clinical prediction model bias risk assessment tool CHARMS list (15). The model was evaluated from 11 aspects: data source, participant, prediction outcome, screening factor, sample size, missing data, model establishment, model performance, model evaluation, result interpretation, and discussion. The quality evaluation of the included literature was completed by two researchers. When there was a disagreement, the third researcher discussed the decision together.

### 2.7 Statistical analysis

Excel software was used to extract the data, and the data were standardized. IBM SPSS Statistics (25.0 version) software was used to analyze the independent risk factors of the infiltration of ground-glass pulmonary nodules, and the order was ranked according to the frequency of occurrence. Review Manager (5.4 version) software was used for the meta-analysis of literature combined with independent risk factors. The heterogeneity test was performed by using *Q* test or *I* ^2^ test. The fixed effect model or random effect model was used to calculate the MD value and 95%CI interval of the combined continuous variables and the OR value and 95%CI interval of the categorical variables. If the heterogeneity test *P <*0.10 or *I* ^2^ > 50% suggests that there is significant heterogeneity in the literature, the random effect model is used; otherwise, the fixed effect model is used and shows the results of a subgroup analysis with risk factors ≥4 times. If the heterogeneity is large, sensitivity analysis is performed, and publication bias is expressed in a funnel plot. *P <*0.05 was considered statistically significant.

## 3 Results

### 3.1 Literature search results

**Figure 1:**
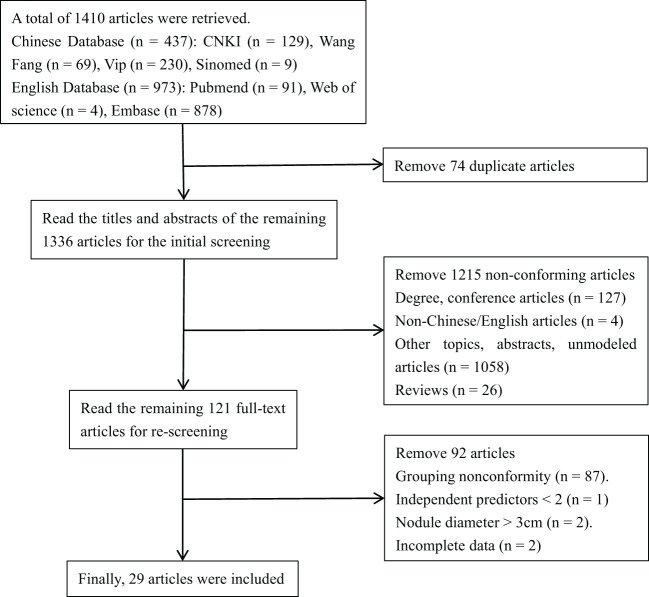
Literature screening flowchart.

A total of 1,410 articles were retrieved, including CNKI (129 articles), Wang Fang (69 articles), Vip (230 articles), Sinomed (nine articles), Pubmed (91 articles), Web of Science (four articles), and Embase (878 articles). By reading the title, abstract, and full text, the repetitive and non-compliant articles were eliminated. Finally, 29 articles were included (16–44). The flowchart of literature screening is shown in **Figure 1**.

### 3.2 Independent risk factors

The independent risk factors of invasive lung cancer in 29 literature were summarized, and the frequency was counted. There were 30 independent risk factors involved in the 29 literature models, with a total of 99 times. There were 16 independent risk factors with a frequency of ≥2 times, with a total of 85 times. The independent risk factors in 29 literature are shown in **Table 1**.

**Table 1:** Frequency distribution of independent risk factors.

Number	Independent risk factors	Frequency
1	Average CT value	14
2	Maximum diameter	13
3	Vascular bundle sign	11
4	Lobulation sign	7
5	Air bronchogram	6
6	Mean diameter	6
7	Maximum CT value	5
8	Spiculation sign	4
9	Volume	3
10	Vacuolar sign	3
11	CTR	3
12	Vascular type	2
13	Pleural indentation	2
14	Irregular shape	2
15	Age	2
16	mGGN	2
17	Maximum diameter of solid component	1
18	Enhancement value	1
19	relative CT value	1
20	Meniscus sign	1
21	Average diameter of solid components	1
22	Volume of the solid component	1
23	Male	1
24	Spiculation or lobulation sign	1
25	ProGRP	1
26	NSE	1
27	NIU-cAF	1
28	EGFR mutation	1
29	Cyfra21-1	1
30	Quality	1

### 3.3 Model characteristics

Finally, 29 articles were included, all of which were published in the past 5 years. Of the 29 studies, 28 were retrospective studies and one was retrospective and prospective study (retrospective modeling and prospective validation), one study conducted internal and external verification, one study conducted external and external verification, six studies conducted internal verification, one study conducted external verification, one study conducted verification (internal and external unknown), and the remaining 19 studies were not verified. There were 27 single-center studies and two multi-center studies. The literature basic characteristics are shown in **Table 2**, and the model prediction efficiency is presented in **Table 3**.

**Table 2:** Basic characteristics of the included studies.

①	②	③	④	⑤	⑥	⑦	⑧	⑨	⑩	⑪	⑫	⑬	⑭	⑮^a^
Chen YM (16)	2023	R	A: 2018.1–2019.12	S	Jiangsu	5 mm–3 cm	T	649	–	292	357	168	481	4, 5, 11, 12, 13
Chen Y (17)	2023	R	A: 2018.1–2022.12	S	Shanxi	≤3 cm	T	193/217	–	144	73	77	116	3, 5, 7, 8
Chou YH (18)	2023	R	A: 2021.7–2021.12	S	Beijing	<2 cm	F	165	–	62	103	45	120	21, 22
Fei J (19)	2021	R	A: 2016.1–2019.10	S	Beijing	≤3 cm	F	188	–	109	79	55	133	1, 6
Jin GY (20)	2022	R	A: 2017.11–2021.3	S	Henan	<3 cm	F	424/447	–	160	287	115	309	1, 2
Li CY (21)	2022	R	A: 2021.2–2021.8	S	Jiangsu	≤3 cm	T	150	–	79	71	42	108	3, 11, 24, 27
Li CY (22) ^b^	2022	R	A: 2021.1–2022.5	S	Jiangsu	6 mm–3 cm	T	134	–	59	75	44	90	2, 17, 18
Li M (23)	2022	R	A: 2019.11–2020.12	S	Tianjin	≤3 cm	F	113/119	–	66	53	30	83	1, 2, 3
Lin C (24)	2022	R	A: 2021.5–2022.5	S	Shanghai	–	F	115	–	50	65	38	77	6, 7
Min XH (25)	2021	R	A: 2019.4–2019.12	S	Anhui	≤3 cm	F	191/196	–	128	68	71	120	1, 2, 3, 5
Xu DX (26)	2023	R	A: 2010.1–2012.1	S	Zhejiang	≤3 cm	T	157	–	89	68	–	–	2, 4, 8, 19
Xu XY (27)	2021	R+P	A: 2015.5–2020.9; B: 2020.10–2021.5	S	Xinjiang	≤3 cm	F	595	B: 250	231	364	279	316	1, 2, 9, 25, 26, 28, 29
Yang XG (28)	2021	R	A: 2015.1–2018.5	S	Guangxi	–	F	150	–	95	55	71	79	1, 2, 4, 5
Yang YT (29)	2024	R	A: 2020.9–2022.7	S	Yunnan	–	T	555	B: unknown	310	245	157	398	1, 2, 3, 7, 8, 11
Yu Y (30)	2020	R	A: 2016.8–2019.10	S	Shanghai	8 mm < ∼30 mm	F	148	–	98	50	33	115	1, 3, 5
Zhang R (31)	2023	R	A: 2018.1–2021.5	S	Guangdong	–	F	207	–	101	106	50	157	6, 20
Zhao L (32)	2020	R	A: 2015.1–2017.1	S	Liaoning	–	F	278	–	92	186	88	190	1, 2
Feng H (33)	2023	R	A: 2017.1–2020.12; B, C: 2017.1–2020.12	S	Hebei	<3 cm	F	232	B: 98; C: 52	172	60	74	158	1, 15, 23
Lv Y (34)	2022	R	A: 2016.1–2021.9	S	Jiangsu	5 mm–3 cm	F	182/216	B: unknown	164	52	56	160	2, 3, 4, 5, 10, 13, 14, 16
Liu J (35)	2022	R	A: 2018.3–2020.12; B: 2019.2–2020.12	S	Sichuan	4–25 mm	T	160	B: 63	96	64	54	106	9, 12
Hu F (36)	2021	R	A: 2017.1–2017.12; B: 2018.7–2018.12	M	Shanghai	≤3 cm	T	344	B: 345	211	133	98	246	3, 4, 6, 7, 8, 10
Chen W (37)	2021	R	A: 2014.1–2018.8	S	Shanghai	≤10 mm	T	318	–	254	64	106	212	3, 14, 16
Xu F (38)	2020	R	A: 2015.1–2017.10; B: 2015.1–2017.10	S	Zhejiang	≤3 cm	T	258	B: 64	120	138	70	188	1, 6, 15
Hong MP (39)	2024	R	A: 2017.8–2022.8; B, C: 2017.8–2022.8	M	Guangdong	–	F	230	B: 157; C: 156	103	127	65	165	2, 4
Zheng H (40)	2022	R	A: 2017.7–2020.12; B: 2017.7–2020.12	S	Hubei	<3 cm	F	219	B: 93	86	133	149	70	3, 4, 6
Li Y (41)	2022	R	A: 2018.7–2020.1; B: 2020.1–2020.12	S	Sichuan	5–30 mm	T	103	B: 44	60	43	67	36	1, 9, 10
Fu J (42)	2023	R	A: 2020.1–2021.12	S	Hubei	5–30 mm	T	89/94	–	35	59	32	57	1, 30
Wang SQ (43)	2020	R	A: 2017.12–2019.3	S	Jiangsu	6 <∼30 mm	F	78/87	–	25	62	24	54	1, 2, 3
Xie YM (44)	2023	R	A: 2019.3–2022.4	S	Anhui	≤2 cm	T	80/90	–	42	48	25	55	2, 7

①, included in the study; ②, published time; ③, research type; ④, research time; ⑤, research center, ⑥, region; ⑦, diameter; ⑧, grouping situation; ⑨, modeling group/nodule; ⑩, validation group/nodule; ⑪, non-infiltration; ⑫, infiltration; ⑬, male; ⑭, female; ⑮, independent risk factors; R, retrospective; P, prospective; A, modeling data set; B, C, validation data set; S, single center; M, multi-center; T, AIS + MIA group, IAC group; F, AAH + AIS + MIA group, IAC group; -, no or missing data.

a Independent risk factors in **Table 1**.

b Reference 22 (same below).

**Table 3:** Model prediction efficiency.

①	②	③	④	⑤	⑥	⑦	⑧	⑨	⑩	⑪	⑫
Chen YM (16)	Logistic regression	–	Hosmer–Lemeshow	5	0.853	–	–	72.3	82.2	–	–
Chen Y (17)	Logistic regression	–	–	4	0.96	0.46	–	89.04	92.36	–	–
Chou YH (18)	Logistic regression	–	–	2	0.849	0.639	81.8	81.6	82.3	–	–
Fei J (19)	Logistic regression	–	–	2	0.884	0.276	–	73.4	89.9	–	–
Jin GY (20)	Logistic regression	–	–	2	0.851	–	–	78.7	80.5	–	–
Li CY (21)	Logistic regression	–	Hosmer–Lemeshow	4	0.889	–	82	93	77.2	–	–
Li CY (22)b	Logistic regression	–	Hosmer–Lemeshow	3	0.913	–	81.3	94.9	77.3	–	–
Li M (23)	Logistic regression	–	–	3	0.909	0.505	–	81.1	86.4	–	–
Lin C (24)	Logistic regression	–	–	2	0.888	–	–	–	–	–	–
Min XH (25)	Logistic regression	–	–	4	0.899	0.196	–	91.2	71.1	–	–
Xu DX (26)	Logistic regression	–	–	4	0.977	–	95.54	97.1	94.4	–	–
Xu XY (27)	Logistic regression	Interior	–	7	0.882/0.863	-/-	-/-	-/-	-/-	-/-	-/-
Yang XG (28)	Logistic regression	–	–	4	0.934	–	–	96.36	81.05	–	–
Yang YT (29)	Logistic regression	Interior	Decision curve Analysis	6	0.910/0.905	0.497/-	-/-	79.2/-	88.4/-	-/-	-/-
Yu Y (30)	Logistic regression	–	–	3	0.814	–	–	90.0	59.18	–	–
Zhang R (31)	Logistic regression	–	–	2	0.807	0.47	–	56.4	90.6	–	–
Zhao L (32)	Logistic regression	–	–	2	0.819	0.682	–	67.74	80.43	–	–
Feng H (33)	Logistic regression	Interior + exterior	Hosmer–Lemeshow	3	0.729/0.652/0.692	-/-/-	63.8/51.0/63.5	81.7/68.0/68.2	57.6/45.2/60.0	-/-/-	-/-/-
Lv Y (34)	Logistic regression	Unknown	–	8	0.78/0.76	-/-	68/67	73/74	67/64	-/-	-/-
Liu J (35)	Logistic regression	Interior	Hosmer–Lemeshow	2	0.873/0.875	-/-	83.1/88.9	84.4/91.7	82.3/87.2	-/-	-/-
Hu F (36)	Logistic regression	Exterior	–	6	0.910/0.883	0.42/0.45	-/-	83.7/81.4	83.9/82.3	-/-	-/-
Chen W (37)	Logistic regression	–	Hosmer–Lemeshow	3	0.736	–	61.6	84.4	62.1	–	–
Xu F (38)	Logistic regression	Interior	–	3	0.853/0.824	-/-	71.88/-	82.35/-	60/-	70/-	75/-
Hong MP (39)	Logistic regression	Exterior + exterior	–	2	0.787/0.759/0.755	0.773/0.660/0.699	73.9/57.3/64.1	80.3/85.5/81.2	66.0/30.9/46.1	-/-/-	-/-/-
Zheng H (40)	Logistic regression	Interior	–	3	0.83/0.78	-/-	79/75	76/72	83/79	87/80	70/71
Li Y (41)	Logistic regression	Interior	Hosmer–Lemeshow	3	0.879/0.897	-/-	84.5/84.1	76.7/73	90/92	-/-	-/-
Fu J (42)	Logistic regression	–	–	2	0.828	0.631	75.5	72.88	82.86	–	–
Wang SQ (43)	Logistic regression	–	Hosmer–Lemeshow	3	0.922	–	88.5	93.6	76	–	–
Xie YM (44)	Logistic regression	–	–	2	0.901	–	–	93.75	71.43	–	–

①, included in the study; ②, modeling method; ③, verification method; ④, model evaluation; ⑤, number of factors; ⑥, AUC (A/B/C); ⑦, P (cutoff point) [A/B/C]; ⑧, accuracy (%) [A/B/C]; ⑨, sensitivity (%) [A/B/C]; ⑩, specificity (%); ⑪, PPV (%) [A/B]; ⑫, NPV (%) [A/B]; A, modeling data set; B, C, validation data set; -, no or missing data; PPV, positive predictive value; NPV, negative predictive value.b Reference 22.

### 3.4 Risk assessment of bias in the included studies

**Figure 2:**
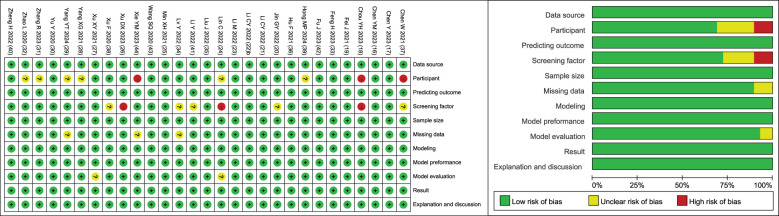
Risk of bias summary graph.3.5 Meta-analysis results.

The included studies were evaluated by the clinical prediction model bias risk assessment tool CHARMS checklist (15). The results showed that the overall bias risk of the included studies was low, and the methodology and quality evaluation were favorable (**Figure 2**).

### 3.5 Meta-analysis results

#### 3.5.1 Analysis of risk factors for infiltration of ground-glass pulmonary nodules

There were 16 independent risk factors that appeared ≥2 times in the 29 articles. CTR appeared in three studies (16, 21, 29), but the classification of CTR in the study (29) was different from that in studies (16, 21). Therefore, the meta-analysis of CTR was temporarily combined studies (16, 21). The results showed that the average CT value, maximum diameter, vascular bundle sign, lobulation sign, mean diameter, maximum CT value, spiculation sign, volume, vacuole sign, CTR, vascular type, pleural indentation, age, and mGGN were risk factors for invasive risk of ground-glass pulmonary nodules. The meta-analysis results are shown in **Tables 4**, **5**. There were eight risk factors that appeared ≥4 times, and we show the results of the subgroup analysis in Sections 3.5.1.1–3.5.1.8.

**Table 4:** Meta-analysis of risk factors of continuous variables.

Risk factors	Number of combined studies	Sample size (*n*)			Heterogeneity test				Meta-analysis results	
		IAC	N-IAC	Total		*I* ^2^ (%)	*P*	EM	MD 95%CI	P
Average CT value (HU)	14 (19, 20, 23, 25, 27–30, 32, 33, 38, 41–43)	1,749	1,701	3,450		94	<0.001	Random	75.57 (44.40, 106.75)	<0.001
Maximum diameter (mm)	13 (20, 22, 23, 25–29, 32, 34, 39, 43, 44)	1,690	1,564	3,254		89	<0.001	Random	4.99 (4.22, 5.77)	<0.001
Mean diameter (mm)	6 (19, 24, 31, 36, 38, 40)	654	677	1,331		80	<0.001	Random	4.46 (3.44, 5.48)	<0.001
Maximum CT value (HU)	5 (17, 24, 29, 36, 44)	564	757	1,321		98	<0.001	Random	112.52 (8.08, 216.96)	0.03
Volume (mm^3^)	3 (27, 35, 41)	471	387	858		0	0.85	Fixed	1069.37 (1025.75, 1112.99)	<0.001
Age (year)	2 (33, 38)	198	292	490		0	0.900	Fixed	4.18 (1.70, 6.65)	<0.001

IAC, infiltration group; N-IAC, non-infiltration group; EM, effects model.

**Table 5:** Meta-analysis of risk factors of categorical variables.

Risk factors	Number of combined studies	Sample size (*n*)			Heterogeneity test			Meta-analysis results	
		IAC (Y/N)	N-IAC (Y/N)	Total	*I* ^2^ (%)	*P*	EM	OR 95%CI	*P*
Vascular bundle sign	11 (17, 21, 23, 25, 29, 30, 34, 36, 37, 40, 43)	731/273	304/1261	2,569	83	<0.001	Random	11.16 (6.71, 18.56)	<0.001
Lobulation sign	7 (16, 26, 28, 34, 36, 39, 40)	580/345	336/704	1,965	94	<0.001	Random	3.80 (1.59, 9.09)	0.003
Air bronchogram	6 (16, 17, 25, 28, 30, 34)	390/265	334/587	1,576	97	<0.001	Random	4.80 (0.87, 26.41)	0.07
Spiculation sign	4 (17, 26, 29, 36)	248/271	108/646	1,273	86	<0.001	Random	4.46 (2.03, 9.81)	<0.001
Vacuolar sign	3 (34, 36, 41)	113/115	65/370	663	76	0.02	Random	6.15 (2.70, 14.01)	0.001
CTR ≥ 0.5	2 (16, 21)	221/207	41/330	799	67	0.08	Random	7.24 (3.35, 15.65)	<0.001
Vascular type (III, IV)	2 (16, 35)	390/31	187/201	809	35	0.22	Fixed	13.62 (8.85, 20.94)	<0.001
Pleural indentation	2 (16, 34)	196/213	67/389	865	81	0.02	Random	6.92 (2.69, 17.82)	<0.001
Irregular shape	2 (34, 37)	74/42	116/208	534	95	<0.001	Random	1.43 (0.19, 10.83)	0.73
mGGN	2 (34, 37)	66/50	111/307	534	0	0.7	Fixed	3.62 (2.36, 5.56)	<0.001

IAC, infiltration group; N-IAC, non-infiltration group; EM, effects model; Y, yes; N, no.

##### 3.5.1.1 Average CT value (HU)

**Figure 3:**
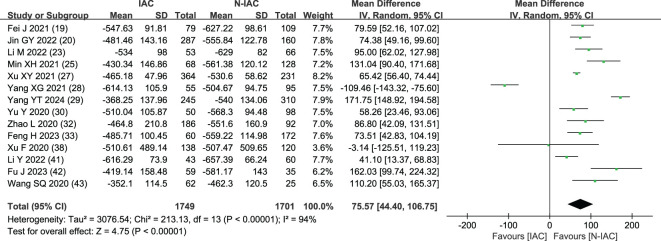
Meta-analysis forest plot of average CT value.

**Figure 4:**
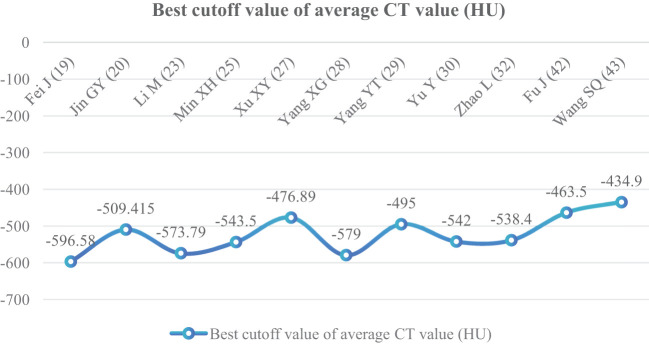
Best cutoff value line chart of average CT value.

In 14 articles (19, 20, 23, 25, 27–30, 32, 33, 38, 41–43), the average CT value of nodules was an independent risk factor, with a total sample size of 3,450 cases. The heterogeneity test showed that there was significant heterogeneity among the studies (*P* < 0.001, *I* ^2^ = 94%); therefore, the random effect model was used. The average CT value of nodules in the infiltration group was larger than that in the non-infiltration group (**Figure 3**). In 11 articles (19, 20, 23, 25, 27–30, 32, 42, 43), the average CT value best cutoff value for predicting IAC nodules was -596.58 to -434.90 HU (**Figure 4**).

##### 3.5.1.2 Maximum diameter (mm)

**Figure 5:**
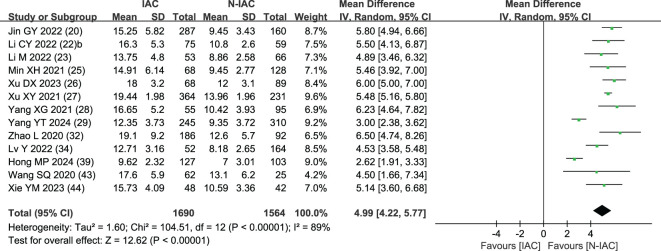
Meta-analysis forest plot of maximum diameter.

**Figure 6:**
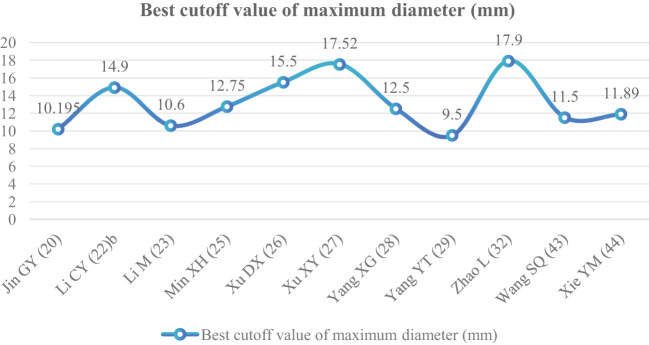
Best cutoff value line chart of maximum diameter.

The maximum diameter of nodules was included as an independent risk factor in 13 articles (20, 22, 23, 25–29, 32, 34, 39, 43, 44). The total sample size was 3,254 cases. The heterogeneity test showed that there was significant heterogeneity among the studies (*P* < 0.001, *I* ^2^ = 89%); therefore, the random effect model was used. The average maximum diameter of nodules in the infiltration group was larger than that in the non-infiltration group (**Figure 5**). A total of 11 articles (20, 22, 23, 25–29, 32, 43, 44) gave the best maximum diameter cutoff value of 9.5–17.9 mm for predicting IAC nodules (**Figure 6**).

##### 3.5.1.3 Vascular bundle sign

**Figure 7:**
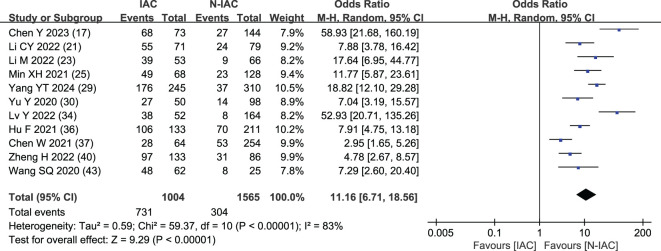
Meta-analysis forest plot of vascular bundle sign.

The vascular bundle sign was included as an independent risk factor in 11 articles (17, 21, 23, 25, 29, 30, 34, 36, 37, 40, 43). The total sample size was 2,569 cases. The heterogeneity test showed that there was significant heterogeneity among the studies (*P* < 0.001, *I* ^2^ = 83%); therefore, the random effect model was used. The probability of vascular bundle sign in the invasive group was significantly higher than that in the non-invasive group (OR = 11.16, 95%CI: 6.71–18.56) (**Figure 7**).

##### 3.5.1.4 Lobulation sign

**Figure 8:**
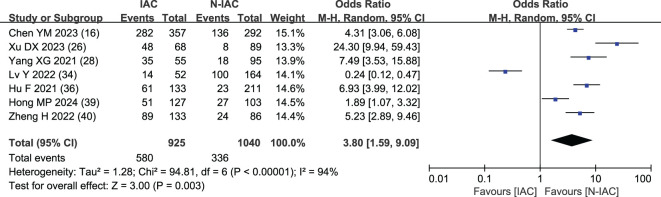
Meta-analysis forest plot of lobulation sign.

The lobulation sign was included as an independent risk factor in seven articles (16, 26, 28, 34, 36, 39, 40). The total sample size was 1,965 cases. The heterogeneity test showed that there was significant heterogeneity among the studies (*P* < 0.001, *I* ^2^ = 94%); therefore, the random effect model was used. The probability of lobulation sign in the infiltrating group was significantly higher than that in the non-infiltrating group (OR = 3.80, 95%CI: 1.59–9.09) (**Figure 8**).

##### 3.5.1.5 Air bronchogram

**Figure 9:**
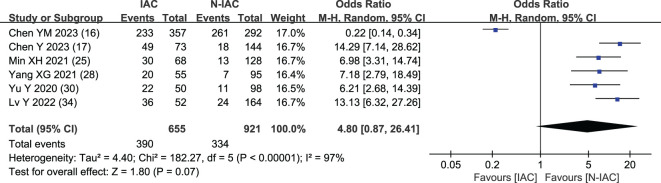
Meta-analysis forest plot of air bronchogram.

In six articles (16, 17, 25, 28, 30, 34), air bronchogram was included as an independent risk factor, with a total sample size of 1,576 cases. The heterogeneity test showed significant heterogeneity among studies (*P* < 0.001, *I* ^2^ = 97%); therefore, the random effect model was used. The meta-analysis showed that there was no significant difference in air bronchogram between the infiltration group and the non-infiltration group (*P* = 0.070 > 0.05), but the probability of air bronchogram in the infiltration group was higher than that in the non-infiltration group (OR = 4.80, 95%CI: 0.87–26.41) (**Figure 9**).

##### 3.5.1.6 Mean diameter (mm)

**Figure 10:**
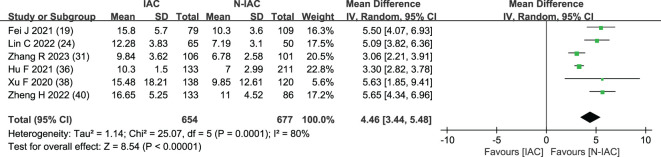
Meta-analysis forest plot of mean diameter.

**Figure 11:**
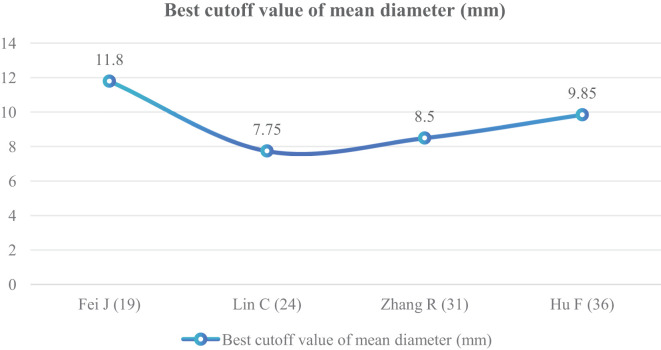
Best cutoff value line chart of mean diameter.

The mean diameter of nodules was included in six articles (19, 24, 31, 36, 38, 40) as an independent risk factor. The total sample size was 1,331 cases. The heterogeneity test showed that there was significant heterogeneity among the studies (*P* < 0.001, *I* ^2^ = 80%); therefore, a random effect model was used. The meta-analysis showed that the mean diameter of nodules in the infiltration group was larger than that in the non-infiltration group (**Figure 10**). The mean diameter cutoff value for predicting IAC nodules in four articles (19, 24, 31, 36) was 7.75–11.8 mm (**Figure 11**).

##### 3.5.1.7 Maximum CT value (HU)

**Figure 12:**
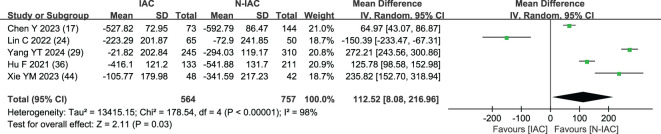
Meta-analysis forest plot of maximum CT value.

**Figure 13:**
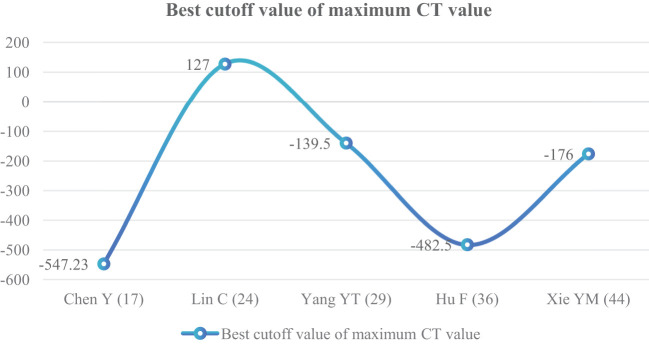
Best cutoff value line chart of maximum CT value.

The maximum CT value of nodules was included in five articles (17, 24, 29, 36, 44) as an independent risk factor, with a total sample size of 1,321 cases. The heterogeneity test showed that there was significant heterogeneity among the studies (*P* = 0.03 < 0.05, *I* ^2^ = 98%); therefore, a random effect model was used. The meta-analysis showed that the maximum CT value of nodules in the infiltration group was larger than that in the non-infiltration group (MD = 112.52 HU, 95%CI: 8.08–216.96) (**Figure 12**). Five articles all gave the maximum CT value cutoff value of -547.23–127 HU for predicting IAC nodules (**Figure 13**).

##### 3.5.1.8 Spiculation sign

**Figure 14:**
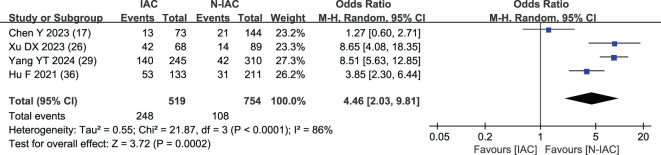
Meta-analysis forest plot of spiculation sign.

Four studies (17, 26, 29, 36) included spiculation sign as an independent risk factor, with a total sample size of 1,273 cases. The heterogeneity test showed that there was significant heterogeneity among the studies (*P* < 0.001, *I* ^2^ = 86%); therefore, the random effect model was used, and the probability of spiculation sign in the infiltration group was significantly higher than that in the non-infiltration group (OR = 4.46, 95%CI: 2.03–9.81) (**Figure 14**).

#### 3.5.2 Sensitivity analysis

Two models of fixed effect and random effect were used to analyze the risk factors with large heterogeneity. The results showed that the maximum CT value and pleural indentation sign were significantly different in the two models, and the results of the other risk factors were stable (**Tables 6**, **7**).

**Table 6:** Sensitivity analysis of risk factors of continuous variables.

Risk factors	Random effect model	Fixed effect model
	Consolidated MD (95%CI)	Consolidated MD (95%CI)
Average CT value (HU)	75.57 (44.40, 106.75)	72.47 (65.99, 78.95)
Maximum diameter (mm)	4.99 (4.22, 5.77)	4.88 (4.66, 5.10)
Mean diameter (mm)	4.46 (3.44, 5.48)	3.75 (3.39, 4.12)
Maximum CT value (HU)	112.52 (8.08, 216.96)	131.33 (117.11, 145.55)

**Table 7:** Sensitivity analysis of risk factors of categorical variables.

Risk factors	Random effect model	Fixed effect model
	Consolidated MD (95%CI)	Consolidated MD (95%CI)
Vascular bundle sign	11.16 (6.71, 18.56)	10.30 (8.49, 12.50)
Lobulation sign	3.80 (1.59, 9.09)	3.44 (2.83, 4.18)
Spiculation sign	4.46 (2.03, 9.81)	5.27 (4.04, 6.44)
Vacuolar sign	6.15 (2.70, 14.01)	5.63 (3.87, 8.20)
CTR ≥ 0.5	7.24 (3.35, 15.65)	5.36 (3.83, 7.50)
Pleural indentation	6.92 (2.69, 17.82)	6.13 (4.05, 9.28)

#### 3.5.3 Publication bias

**Figure 15:**
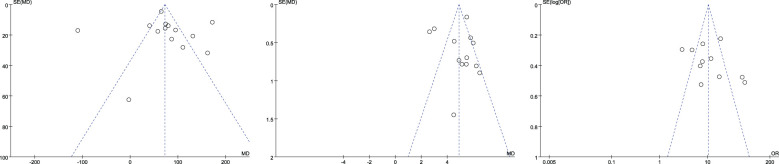
Mean CT value (left), maximum diameter (between), and vascular bundle sign (right) funnel plot.

The funnel plot was used to test the publication bias of the included literature of more than nine articles. The average CT value, maximum diameter, and vascular bundle sign were included in the literature of more than nine articles. Their funnel plots were basically symmetrical, and there was no obvious publication bias. The funnel plots are shown in **Figure 15**.

## 4 Discussion

Ground-glass pulmonary nodules are common lung imaging findings, with a high incidence in the population, especially in the wake of the COVID-19 pandemic as the use of chest CT scans has surged (45). The proportion of ground-glass pulmonary nodules in patients with lung cancer surgery has gradually increased. It has been found that the proportion of ground-glass pulmonary nodules in patients with lung cancer surgery has increased from 18.6% in 2016 to 57.3% in 2019, while the proportion of solid pulmonary nodules has decreased (46). This phenomenon has broken the traditional concept that lung cancer is mostly a solid lesion, and ground-glass pulmonary nodules have gradually become the main culprit of lung cancer. Moreover, 70%–90% of persistent ground-glass pulmonary nodules are lung cancer, and of GGNs that persisted for 3 to 4 months after follow-up, 60%–80% were pre-invasive or invasive lesions (47–49). A study in Japan found that the postoperative pathology of ground-glass pulmonary nodules was, in all cases, adenocarcinoma (50). The pathological morphology of ground-glass pulmonary nodules often undergoes atypical adenomatous hyperplasia (AAH) → adenocarcinoma *in situ* (AIS) → minimally invasive adenocarcinoma (MIA) → invasive adenocarcinoma (IAC) (51), even in patients with EGFR mutations (52).

The treatment strategies and prognosis of ground-glass pulmonary nodules at different stages are different (53). AAH, AIS, and MIA mostly undergo segmentectomy, the prognosis is good, and the 5-year survival rate was nearly 100%. IAC often requires lobectomy, the prognosis is poor, and the 5-year survival rate decreased to 73%–90% (54). The early and accurate diagnosis of GGNs has great significance to prevent overtreatment and alleviate the suffering of patients (55). As ground-glass nodules have a stepwise progression in lung adenocarcinomas, in recent years, the risk prediction models of ground-glass pulmonary nodules have emerged in an endless stream, such as benign/malignant, invasive/non-invasive (56–58). It has become a hot issue in current research and discussion and has also been proposed as a potential means to overcome the limitations of size-based uncertainty in the risk assessment of malignant lung nodules (59). As ground-glass pulmonary nodules are mostly inert cancers, studies have shown that more than 90% of ground-glass pulmonary nodules are stable during 4 to 5 years of follow-up (60, 61); thus, early identification of invasive ground-glass pulmonary nodules, grasping the opportunity of intervention, actively taking treatment measures, and selecting appropriate surgical methods are of great significance for the prevention and treatment of lung cancer and improving the prognosis.

Although the existing prediction models are helpful for IAC identification, the predictors for distinguishing IAC and N-IAC are different in each study, so it is necessary to carry out further meta-analysis research. Since logistic regression model is the most common modeling method, the independent predictors can be obtained (62). This study systematically evaluated and meta-analyzed the logistic regression risk prediction models of ground-glass pulmonary nodules at home and abroad through literature review, explored the independent risk factors of ground-glass pulmonary nodule infiltration risk and the influence of the combination of independent factors on the infiltration of ground-glass pulmonary nodules, and analyzed the prediction efficiency of the model. It was found that the overall performance of the prediction model is good, and the verification and extrapolation still need to be further studied and discussed. This study can provide a reference for the identification of infiltration of ground-glass pulmonary nodules.

### 4.1 Analysis of independent risk factors

The results of the meta-analysis of independent risk factors showed that average CT value, maximum diameter of nodules, vascular bundle sign, lobulation sign, air bronchogram, mean diameter of nodules, maximum CT value, spiculation sign, volume, vacuole sign, CTR ≥0.5, vascular type (III and IV), pleural indentation sign, irregular shape, mGGN, and age in traditional features were risk factors for the prediction of invasive risk of ground-glass pulmonary nodules by logistic regression. Knowing the risk factors of IAC is helpful to guide clinical decision-making and treatment. The consolidated sample size of meta-analysis was ≥490 cases in all.

#### 4.1.1 Meta-analysis of continuous variables

The meta-analysis of continuous variables showed that the average CT value, maximum diameter, mean diameter, maximum CT value, volume, and age of GGNs patients were risk factors for its invasion.

The average CT value reflects the thickening of myofibroblast matrix caused by invasive tumor cell infiltration (63). The high average CT value shows the proliferation of a large number of tumor cells in the interstitial tissue (64); in other words, the strong invasive ability reflects the higher average CT value. The study had shown that the average CT value of IAC was higher than that of AAH/AIS/MIA, the subtype of IAC had the lowest average CT value in wall growth type, and the average CT value of acinar IAC was lower than that of papillary IAC (65). Our study found that the average CT value was closely related to the infiltration of nodules, and it appeared as the most main independent predictor in 14 models (19, 20, 23, 25, 27–30, 32, 33, 38, 41–43), which means that it plays an important role in the identification of IAC and N-IAC. The meta-analysis showed that the average CT value in the IAC group is higher than that in the N-IAC group (MD = 75.57 HU, 95%CI: 44.40–106.75). The mean range of the average CT value in the IAC group was -616.29 to -352.10 HU, while in the N-IAC group it was -657.39 to -462.30 HU. It means that when the average CT value is higher than -462.30 HU, GGNs may mostly be IAC, and this is consistent with the study findings that nodules are more likely to be invasive adenocarcinoma when pGGNs have higher pixel attenuation than -472 HU (47). Among the 14 models, 11 models (19, 20, 23, 25, 27–30, 32, 42, 43) had given the best cutoff values of the average CT value to distinguish IAC and N-IAC within the range of -596.58 to -434.90HU. When the critical value was -579 HU, the AUC value of IAC identified from GGNs was best at 0.934. When the critical values were -573.79, -495, and -434.9 HU, it still had higher IAC and N-IAC discrimination ability (AUC: 0.909–0.922). This suggests that when the average CT value is higher than -434.9 HU, GGNs are more likely to be IAC is beyond doubt. However, the difference in the cutoff value of -596.58 to -434.9 HU may be related to the different subtypes of IAC included. The pathology subtype of the IAC includes LPA, APA, and PPA. Among them, papillary IAC is more prone to vascular infiltration or lymphatic infiltration and pleural infiltration or cell airway diffusion or necrosis (66). Therefore, further subtype analysis of IAC to determine the average CT cutoff value of different subtypes is also a future research direction. In addition to the average CT value, the maximum CT value is also a significant indicator for predicting GGNs as IAC. This study shows that the maximum CT value has a large heterogeneity and ranges from -547.23 to 127 HU, which may be due to the fact that the measurement of the maximum CT value is easily affected by the internal structural characteristics and region of interest of the nodule (67). Therefore, the average CT value should still be focused on when predicting IAC.

Although GGNs grow slowly and more image features except diameter are used to distinguish IAC and N-IAC in GGNs, the size is still the key, and there has been a positive correlation between nodule size and tumor (59). The maximum diameter of nodules in 13 prediction models (20, 22, 23, 25–29, 32, 34, 39, 43, 44) was the closest factor related to the infiltration of nodules. The maximum diameter of nodules refers to the long diameter of the maximum diameter cross-section of nodules in lung CT. The greater the maximum diameter of GGN, the higher the possibility of IAC (68). Of course, at times irregularly shaped elongated pulmonary nodules may be benign scar nodules or fibropathy such as pulmonary adenofibroma (69), so in some cases only focusing on the maximum diameter has some limitations. We find that the average maximum diameter of the IAC group was higher than that of the non-IAC group (MD = 4.99, 95%CI: 4.22, 5.77). This may be related to the increase of tumor invasiveness and structural changes. A total of 11 models (20, 22, 23, 25–29, 32, 43, 44) had given the best cutoff value of prediction of IAC maximum diameter. The cutoff value of maximum diameter for predicting IAC was 9.5–17.9mm; 15.5 mm was the best cutoff value of maximum diameter, and it had the best AUC value of 0.977 to distinguish IAC. This means that mostly all of the maximum diameter values greater than 15.5 mm are IAC. When the cutoff values were 14.9, 10.6, 12.5, 9.5, 11.5, and 11.89 mm, the model also had good IAC prediction performance, and the AUC value was 0.901–0.934, while in other cutoff values the AUC value was 0.819–0.899. Although the maximum diameter of pulmonary nodules is closely related to IAC, we found that different studies have different cutoff values of the maximum diameter when distinguishing IAC and had a large range of 8.4 mm (29, 32). In recent years, the measurement of diameter in lung cancer screening is mainly based on the mean diameter (the mean value of the maximum cross-sectional long diameter and the vertical short diameter of the long diameter) (70). Therefore, it is also necessary to pay attention to the mean diameter when distinguishing IAC and N-IAC, especially for irregular nodules with a large difference between the long diameter and the short diameter. It was found that the average diameter of the IAC group was 10.81 mm, and the average diameter of the N-IAC group was 8.54 mm (65). In our study, the average diameter as a risk factor appeared in six articles (19, 24, 31, 36, 38, 40), of which four articles (19, 24, 31, 36) gave the best cutoff value range to be from 7.75 to 11.8 mm. This indicates that GGNs with a mean diameter of the nodule ≥7.75 mm should also be alert to the possibility of ICA. Compared with the maximum diameter, the mean diameter predicts that the IAC best cutoff value fluctuates relatively small. Therefore, the clinical diagnosis of IAC should be based on the average diameter and also combined with the maximum diameter.

Beyond diameter and CT value, volume and age were also risk factors for GGNs as IAC. Three studies (27, 35, 41) showed that GGN volume was an independent risk factor for infiltration, and the consolidated MD value was 1,068.47 mm (3). Xu’s (27) study showed that the predicted volume best cutoff value of IAC was 1,840.18 mm^3^. The study had found that the nodule volume gradually increased with the increase of GGN infiltration, and the average volume of the IAC group was 1,807.72 mm^3^ (71). Volume changes of GGNs are often used to predict the growth and prognosis of lung cancer and could be divided into three types: fast, medium, and slow growth, in which CT screening has the least reduction in mortality of slow growth (72). Age is the main attention factor for lung cancer screening. The risk of GGN infiltration increased with age. In two studies (33, 38), age was included as one of the prediction risk factors for IAC, indicating that the risk of IAC in ground-glass nodules of different ages in the same imaging performance cannot be generalized—for example, GGNs of 10 mm in 50-year-old patients may be AIS or MIA, while in 55-year-old patients they may be IAC.

The meta-analysis of categorical variables showed that vascular bundle sign, lobulation sign, spiculation sign, vacuolar sign, CTR ≥0.5, vascular type (III and IV), pleural indentation, and mGGN were risk factors for GGN invasion.

Vascular bundle sign is a sign of looting blood nutrition and vascular metaplasia during the growth and infiltration of cancer cells. It is easier to detect than solid components and is common in IAC, as a potential feature of tumor growth in IAC, and with the increase of IAC pathological subtypes, the vascular bundle sign gradually increased (73). In our study, the OR value of vascular bundle sign in the IAC group vs. the non-IAC group was 11.16, consistent with previous studies. Chen’s study (17) included the AUC value of the prediction model of vascular bundle sign as the highest at 0.96, and the sensitivity and specificity were both >89%, which showed the nice prediction efficiency.

The lobulation sign, especially the depth and moderate lobulation sign, is closely related to IAC (74). With the increase of GGN invasion, tumor cells and normal tissue growth imbalance will appear as lobulation sign. It is the main factor to distinguish IAC and N-IAC. In seven articles, the lobulation sign appeared as an independent predictor of IAC with AUC value of 0.78–0.977, and its merged OR value in the IAC group and the N-IAC group was 3.80. The highest AUC value of lobulation sign is higher than that of the vascular bundle sign and has a fine predictive performance (AUC > 0.75). It is necessary to focus on the influence of lobulation depth on IAC in the future.

Spiculation sign is a linear shadow formed by the extension of pulmonary nodules along the edge of the lesion to the lung parenchyma. With the increase of GGN infiltration, the growth rate of tumor cells was accelerated, and the growth of tumor cells was not uniform within the surrounding tissues, resulting in the growth of tumor cells in all directions and in the spiculation sign. It is an important indicator to distinguish benign and malignant and invasive GGNs (75). Spiculation sign can also appear in some inflammatory lesions, such as peripheral pulmonary nodules formed by inflammatory exudation of pulmonary tuberculosis that may be accompanied by spiculation sign (76), which may affect the diagnosis IAC of GGNs to a certain extent. We found that the spiculation sign was significantly higher in the IAC group than in the N-IAC group, with an OR value of 4.46. It prediction of GGNs as IAC had a high AUC value from 0.91 to 0.977 (>0.90). Lobulation sign and spiculation sign are always combined to predict IAC. Xu’s study (26) combined lobulation sign and spiculation sign in predicting IAC that showed the high value of AUC at 0.977.

The vacuole sign is closely related to the rapid growth of tumor tissue and the emptying of necrotic tissue. Vacuole sign is a risk factor for malignant lung cancer, which is more common in grades 1 to 2 invasive lung cancer (77). In our study, vacuole signs appeared as predictors of infiltration risk in three articles (34, 36, 41), with a merge OR at 6.15, which was higher than that in the lobulation sign and the spiculation sign. The AUC value of IAC predicted by Lv’s article was only 0.78; therefore, in order to improve the predictive efficiency, the vacuole sign should be combined with other CT signs.

As a risk factor for the malignant risk of GGNs, the pleural indentation sign also plays an important role in the risk of invasion, especially in IAC near the pleura. The pleural indentation sign is usually a dent or defect between the pleura formed by the tumor or chronic chest inflammation. Since this sign is not unique to IAC, we found that the sensitivity and specificity of pleural indentation in predicting IAC were not particularly high (<0.85), especially in model validation, so the prediction of GGNs as IAC risk should also be combined with other CT infiltration features. Hu’s study (36) showed that any of the five CT signs (vascular bundle, lobulation, spiculation, pleural indentation, and vacuole sign) showed that the risk of IAC was higher, indicating that the risk of IAC should be obvious when the abovementioned signs appear. Furthermore, the composition near the pleura is also closely related to IAC. Studies have shown that IAC of acinar/papillary near the pleura is more common (78).

CTR ≥0.5 was included as an independent risk factor in two studies (16, 21). CTR was the ratio of the maximum diameter of the solid component of the lung window to the maximum diameter of the nodule (the ratio of the solid component, 0–1). The OR value of CTR was ≥0.5 in the IAC group and 7.24 in the N-IAC group. CTR had a significant impact on the degree of infiltration and prognosis, which was only second to vascular bundle sign. The smaller the CTR, the lower the degree of infiltration, the lower the probability of postoperative recurrence, and the higher the 5-year survival rate (79), indicating that the proportion of solid components should be measured when performing lung CT examination for GGN with solid components and that attention should be given to CTR ≥0.5 nodules and increased CTR values of nodules. In addition, Yang’s study (29) considered that CTR ≥0.235 was a risk factor for predicting IAC, and the CTR threshold was relatively lower.

With the increase of tumor invasion, blood vessels will continue to regenerate, as well as in GGNs, and different types of blood vessels will appear. Chen’s study (16) and Liu’s study (35) divided the vascular typing characteristics of GGN into four types: type I—no internal penetrating blood vessels, adjacent edges only marginal blood vessels; II—only one through the blood vessels, walking naturally, the diameter of the normal or thickening; III—≥2 perforating blood vessels, running naturally, with normal or thickened diameter; IV—≥2 perforating vessels, unclear structure, with thickening or reticular anastomosis, among which types III and IV vascular typing are more common in IAC (OR = 13.62). Chen’s study (16) showed that the AUC value and specificity of the prediction model with vascular typing were higher than those of the prediction model without vascular typing. The study of vascular classification in IAC of GGNs should be the focus of future research. The positional relationship between blood vessels and nodules will also affect the choice of treatment methods.

With the increase of the degree of infiltration of pulmonary nodules, the density of pulmonary nodules gradually becomes uneven, and more pGGN becomes mGGN. mGGN is a risk factor for the infiltration of ground-glass pulmonary nodules. A study (80) shows that about 25% of mGGN ≤10 mm is IAC, and about 50% of mGGN >10 mm is IAC. This study shows that the OR value of mGGN in the IAC group and the N-IAC group is 3.62, which is consistent with the existing study results. Beyond mGGN, the maximum diameter of the solid component in GGNs is also an indispensable risk factor for IAC, especially the solid component ≥6 mm, which has been recognized as an indicator for distinguishing IAC and N-IAC, and also a newly revised threshold standard for T staging of adenocarcinoma (81).

In addition to the abovementioned risk factors, new biological indicators should also be paid attention to such as tumor markers (such as abnormal tumor markers or threshold of tumor markers) and the role of mutant genes in the development of GGNs into IAC. These will be the focus of future research directions.

### 4.2 Model establishment, evaluation, and prediction efficiency analysis

All of the models included in this study were based on logistic regression. Multivariate logistic regression is a common independent factor screening and modeling method. The influence of linear variables can be excluded when modeling, and the selected factors are usually independently related to the outcome indicators. The *P*-value of IAC risk probability is usually calculated according to the weight of regression coefficient and the assignment of independent variables when predicting the risk of invasion. A total of 11 studies (17–19, 23, 25, 29, 31, 32, 36, 39, 42) obtained the best cutoff value of GGN invasion risk. According to the best cutoff value of the model, the possibility of IAC of nodules can be determined. The risk probability *P*-value higher than the best cutoff value is always IAC, while if it is lower than the best cutoff value it is usually N-IAC. The prediction efficiency of the prediction model is simply expressed by the area AUC value under the ROC curve (value 0–1), when close to 1, indicating that the higher the prediction efficiency of the model, the better the prediction performance of the model included in this study. To be specific, when AUC is between 0.7 and 0.8, this indicates that the model has medium prediction results, between 0.8 and 0.9 the prediction result is better, and between 0.9 and 1.0 the prediction result is excellent. The AUC value of the modeling group is 0.736–0.977, all >0.70, which shows the upper-middle prediction ability, and most AUC values of the validation group (27, 29, 34–36, 38–41) are >0.70, indicating that most models still have upper-middle performance in validation and can be used to identify the infiltration of GGNs.

In this study, 10 models (27, 29, 33–36, 38–41) were validated after modeling. Six models (27, 29, 35, 38, 40, 41) were internally validated. The AUC value of internal validation was 0.78–0.905, indicating that the internal performance of the model was good, but its extrapolation was not clear. One model (36) was externally validated, and the AUC value of the external validation was 0.883, indicating that the model still had good predictive power when extrapolated. One model (33) was subjected to internal and external validation, but its AUC values for internal and external validation were 0.652 and 0.692, respectively, indicating that the predictive efficacy of internal replication or extrapolation was to be discussed. Although one model (34) was verified, and the AUC value of the verification group was 0.76, which was similar to the 0.78 of the modeling group, the source of the verification data was unknown, and the risk of promotion was not clear. The remaining 19 models were not validated, which may be related to the fact that the model included in this study is an invasive prediction model. Limited research population, relatively single research center, relatively small sample size of IAC, and long time to carry out prospective validation studies have led to these studies not being able to conduct internal or external validation so that they still need future in-depth study when used internally or promoted externally.

In the included studies, nine models (16, 21, 22, 29, 33, 35, 37, 41, 43) were evaluated. Hosmer–Lemeshow test was performed on eight models, which showed that the model fits well. The decision curve analysis of one model (29) showed that the prediction model was suitable for clinical decision-making. The quality of the literature was evaluated with the CHARMS list, which showed that the overall bias risk of the included studies was low, and the research results had a certain clinical reference value.

### 4.3 Limitation analysis of the study

(1) The models included in this study are mostly imaging feature models or clinical models combined with traditional features. Considering that there are many parameters, large differences, high difficulties, and limited applicability of emerging models such as radiomics and random forest., this study is not included, yet attention should be paid to these fields in the future. (2) This study is mostly a retrospective study based on hospital cases or imaging data, and there are few prospective case–control studies. (3) There are relatively few references for some factors, such as gender, tumor markers, etc., which cannot be sub-combined and meta-analyzed. Some factors cannot be analyzed for publication bias due to the lack of literature. (4) There may be some selection bias in literature retrieval and inclusion in that only Chinese and English studies are selected.

## 5 Conclusion

In this study, a systematic evaluation of the risk prediction model of ground-glass pulmonary nodules was conducted, and a meta-analysis of the independent predictors in the logistic regression model was conducted. It was found that average CT value, maximum diameter, vascular bundle sign, lobulation sign, mean diameter, maximum CT value, spiculation sign, volume, vacuolar sign, CTR ≥0.5, vascular type (III and IV), mGGN, and age were risk factors for the infiltration risk of ground-glass pulmonary nodules. The included models had good predictive efficacy. The independent risk factors and prediction models selected in this study and their optimal best cutoff values can help clinical medical workers identify the high-risk population of IAC in ground-glass pulmonary nodules. Thus, appropriate treatment strategies and surgical methods can be selected to improve the prognosis of patients. However, the use of the predictive models included in this study still needs to be verified further. In the future, models containing biomarkers in in-depth research are still needed to expand the scope of application of the model.

## Data Availability

The original contributions presented in the study are included in the article/supplementary material. Further inquiries can be directed to the corresponding author.
